# Mortality risk after cancer diagnosis in kidney transplant recipients: the limitations of analyzing hospital administration data alone

**DOI:** 10.1002/cam4.1367

**Published:** 2018-02-14

**Authors:** Francesca Jackson‐Spence, Holly Gillott, Sanna Tahir, Jay Nath, Jemma Mytton, Felicity Evison, Adnan Sharif

**Affiliations:** ^1^ University of Birmingham Birmingham B15 2TH UK; ^2^ Department of Nephrology and Transplantation Queen Elizabeth Hospital Edgbaston Birmingham B15 2WB UK; ^3^ Department of Health Informatics Queen Elizabeth Hospital Edgbaston Birmingham B15 2WB UK

**Keywords:** Cancer, epidemiology, incidence, kidney transplant, mortality

## Abstract

Administrative data are frequently used for epidemiological studies but its usefulness to analyze cancer epidemiology after kidney transplantation is unclear. In this retrospective population‐based cohort study, we identified every adult kidney‐alone transplant performed in England (2003–2014) using administrative data from Hospital Episode Statistics. Results were compared to the hospitalized adult general population in England to calculate standardized incidence and mortality ratios. Data were analyzed for 19,883 kidney allograft recipients, with median follow‐up 6.0 years' post‐transplantation. Cancer incidence was more common after kidney transplantation compared to the general population in line with published literature (standardized incidence ratio 2.47, 95% CI: 2.34–2.61). In a Cox proportional hazards model, cancer development was associated with increasing age, recipients of deceased kidneys, frequent readmissions within 12 months post‐transplant and first kidney recipients. All‐cause mortality risk for kidney allograft recipients with new‐onset cancer was significantly higher compared to those remaining cancer‐free (42.0% vs. 10.3%, respectively). However, when comparing mortality risk for kidney allograft recipients to the general population after development of cancer, risk was lower for both cancer‐related (standardized mortality ratio 0.75, 95% CI: 0.71–0.79) and noncancer‐related mortality (standardized mortality ratio 0.90, 95% CI: 0.85–0.95), which contradicts reported literature. Although some plausible explanations are conceivable, our analysis likely reflects the limitations of administrative data for analyzing cancer data. Future studies require record linkage with dedicated cancer registries to acquire more robust and accurate data relating to cancer epidemiology after transplantation.

## Introduction

Kidney transplantation is acknowledged as the treatment of choice for people with end‐stage kidney failure, but the need for lifelong antirejection therapy to prevent allograft failure is associated with the development of immunosuppression‐related complications 1. One of the major complications after kidney transplantation is the development of cancer, and the similarity of (infection‐related) cancer development between kidney allograft recipients and patients with HIV/AIDS suggesting immune deficiency, rather than other risk factors, contributes to increased cancer rates after kidney transplantation 2. Cancer is one of the greatest concerns for kidney allograft recipients themselves 3, is associated with increased cost 4, and is linked to increased incidence and mortality in population cohort studies 5, 6, 7, 8, 9, 10, 11, 12, 13. This makes it imperative to understand cancer development, and its risk for progression, if we are to improve long‐term outcomes after kidney transplantation.

Cancer‐related mortality has been shown to be higher among kidney allograft recipients compared to the general population in cohorts from England 6 and Ontario, Canada 13, although equivalent cancer‐related mortality was observed in a US cohort 5. Some methodological considerations limit interpretation of such data. Firstly, analysis of cancer‐related mortality was not always undertaken specifically among the cancer incident cohort. This is important as reporting risk for cancer‐related mortality in isolation will be skewed by increased incidence. Secondly, risk for cancer versus noncancer death among kidney allograft recipients after developing cancer is often overlooked. This is important because increased rates of cancer incidence after transplantation may not directly lead to cancer‐linked mortality as competing risks for death occur after kidney transplantation in the context of immunosuppression (e.g., cardiovascular events or infection).

The use of administrative data for research studies is championed in the setting of solid organ transplantation 14. With ready access to national administration data for secondary care (i.e., hospital) episodes, we were keen to explore the usefulness of utilizing such data to explore cancer outcomes after kidney transplantation. Therefore, we undertook a population cohort study to explore cancer incidence and subsequent progression to mortality (cancer vs. noncancer) among kidney allograft recipients in England compared to the general hospitalized population using national administrative data.

## Materials and Methods

### Study population

We obtained data for every kidney‐alone transplant procedure performed in England between 2003 and 2014, collecting patient demographics that were recorded at the time of admission for kidney transplantation. Data were obtained from Hospital Episode Statistics (HES) 15, an administrative data warehouse containing admissions to all National Health Service hospitals in England. It contains detailed records relating to individual patient treatments, with data extraction facilitated utilizing codes on procedural classifications (Office of Population Censuses and Surveys Classification of Interventions and Procedures, 4th revision [OPCS‐4]) 16 and medical classifications (World Health Organization International Classification of Disease, 10th revision [ICD‐10]) 17.

This study included all kidney‐alone transplant procedures (OPCS‐4 codes; M01) performed over the study time period. Cancer was defined from ICD‐10 codes C00‐C99 (excluding nonmelanoma skin cancer (C44)) post‐transplantation. As HES represents secondary care administrative data, any diagnosis of cancer not involving admission to hospital would not be captured. With regard to outcome analysis, the HES dataset is limited to only capturing deaths occurring in a hospital setting. Therefore, we linked our HES cohort with mortality data from the Office for National Statistics (ONS) 18, which collects information on all registered deaths in the United Kingdom. This study did not require institutional review board approval due to the pseudoanonymized nature of the data retrieved—data were linked by NHS Informatics using deterministic methodology using special HES ID codes and avoided patient identifiable data. We registered this project as an audit with University Hospitals Birmingham NHS Trust (audit identifier; CARMS‐12578). This study is reported in accordance with the RECORD (REporting of studies Conducted using Observational Routinely collected health Data) statement 19.

### Data inclusion

We excluded the following transplant recipients from analysis; missing age or sex, residence outside England, combined solid organ transplant, pretransplant history of cancer, and pediatric cases (aged under 18 years). Our comparative general population cohort comprised of all adults (aged 18 and over) admitted to an English hospital with a diagnosis of cancer, excluding those with a previous history of cancer or a solid organ transplant. Results from HES with less than five cases were not numerically identified and were classed as too small to report to avoid potential patient identifiability (in accordance with data agreement).

### Statistical analysis

Primary outcome measures were cancer incidence after kidney transplantation and risk for cancer versus noncancer mortality. Standardized incidence ratios were obtained using the indirect standardization method, with age‐, sex‐, and cancer‐specific specific rates from ONS life tables of the general population, to measure the risk of cancer incidence in the transplant cohort compared to the general population. Standardized mortality ratios were calculated using cancer patients only from the transplant and general population cohorts. We utilized the indirect standardization method, using age‐, sex‐, and cancer‐specific rates from the general population cohort, to measure the risk of cancer or noncancer mortality in the transplant versus general population cohorts (with 95% confidence intervals). The general population cohort was defined as patients admitted to hospital between 2003 and 2014, with no recorded solid organ transplantation or previous history of cancer.

Unadjusted survival analyses were performed with the generation of Kaplan–Meier plots with log‐rank test used for comparative analysis. We conducted Cox regression analysis to examine risk factors for post‐transplant cancer after adjustment for; age, sex, ethnicity, type of donor, number of readmissions in the first year post‐transplant, delayed graft function, Charlson comorbidity score, and whether they had a repeat kidney transplant or not. A *P*‐value <0.05 was considered statistically significant in the analysis. Statistical evaluations were performed using Stata version 14 (StataCorp LP, College Station, TX).

### Role of the funding source

This work was conducted by authors undertaking an intercalated degree with the University of Birmingham, and they received individual financial bursaries from Kidney Research UK and the Arthur Thomson Trust. The funders had no role in the design, execution, or analysis of this work.

### Data access

All authors have reviewed the final submission, and the corresponding author had final responsibility for the submission of this article. In line with our Data Sharing Agreement with NHS Digital, full access to data remained under the sole responsibility of the Department of Health Informatics.

## Results

### Descriptive analysis for transplant cohort

**Figure 1:**
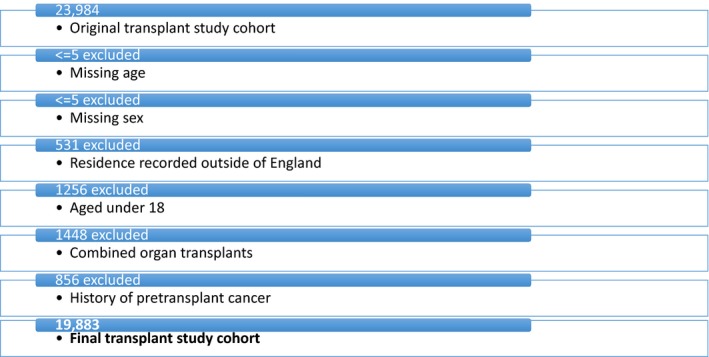
Transplant study cohort showing exclusions.

Between 2003 and 2014, there were 23,984 patients in England who had a kidney transplant recorded in our administrative dataset. After exclusions (Fig. 1), we had a cohort of 19,883 kidney allograft recipients aged 18 and over for analysis. The cohort was followed to December 2015, with median follow‐up time 6.0 years (or 114,569 patient‐years). Table 1 highlights the descriptive statistics of this transplant study cohort.

**Table 1:** Baseline demographics of the English kidney allograft recipient cohort

Variable		Number of Patients
Total number of patients		19,883
Age (years)	Mean (SD)	47.55 (13.69)
Post‐transplant hospital stay (days)	Median (IQR)	7 (5–10)
Sex	Male	12,223 (61.47%)
	Female	7660 (38.53%)
Index of multiple deprivation (multiple deprivation index comprised from; income, employment, health, education, housing, crime, and living environment)	1 (Most deprived)	4530 (22.78%)
	2	4320 (21·73%)
	3	3892 (19.57%)
	4	3547 (17.84%)
	5 (Least deprived)	3480 (17.50%)
	Unknown	114 (0.57%)
Type of donor	Living	7174 (36.08%)
	Deceased	12,381 (62.27%)
	Unknown	328 (1.65%)
Operation year	2003–2006	5008 (25.19%)
	2007–2010	6885 (34.63%)
	2011–2014	7990 (40.19%)
Repeat transplant		807 (4.06%)
Ethnic group	White	15,351 (77.21%)
	Black	1331 (6.69%)
	Other	3201 (16.10%)
Charlson comorbidity score (renal disease excluded)	0	16,194 (81.45%)
	1–4	2231 (11.22%)
	5+	1458 (7.33%)

As our data utilized administration data, we were keen to ensure the data were reflective of actual transplantation activity. We corroborated our numbers with data from the UK Transplant Registry data, where every transplant center has a mandatory requirement to register every kidney transplant (accessed via the UK National Transplant Network). Data corroboration was robust with 95.6% concordance (23,984/25,079) between the administrative and UK Transplant registry datasets, respectively.

### Comparison of post‐transplant cancer incidence

**Figure 2:**
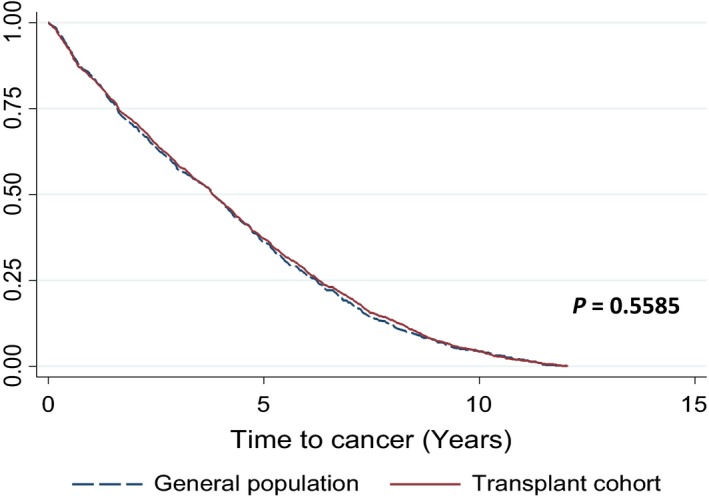
Unadjusted Kaplan–Meier plot of time to cancer.

We identified 1273 kidney allograft recipients (6.40% of the transplant patient cohort) who were admitted with a new cancer diagnosis post–kidney transplantation. The corresponding cancer incident cohort from the general population (with at least one hospitalized episode to an English hospital with a cancer diagnosis during study period) comprised of 1,750,197 persons aged 18 and over, with no previous cancer diagnosis or solid organ transplant. Table 2 shows the raw data for type of cancer comparing the transplant to the general population cohort, highlighting increased risk for selected cancers (e.g., lymphoma, kidney) but decreased risk for others (e.g., breast, prostrate) post‐transplantation. The overall standardized incidence ratio for cancer incidence was 2.47 (95% CI: 2.34–2.61), confirming significantly increased rates of cancer among kidney allograft recipients versus the general population, although rates varied among different cancers (see Table 3). In both the unadjusted Kaplan–Meier curves (see Fig. 2) and the adjusted Cox regression analysis (see Table 4), there was no difference in the time taken to develop cancer between the kidney transplant and general population cohorts.

**Table 2:** Incidence of cancer comparing transplant and general population cohort

Type of cancer category	Transplant cohort *N* (% of total cancers)	General population cohort *N* (% of total cancers)
Bladder	55 (4.3%	46,890 (2.7%)
Brain	9 (0.7%)	37,850 (2.2%)
Breast	81 (6.4%)	200,481 (11.5%)
Cervix Uteri	10 (0.8%)	15,417 (0.9%)
Colon and Rectum	78 (6.1%)	133,080 (7.6%)
Hodgkin's Disease	13 (1.0%)	11,003 (0.6%)
Kidney except Renal Pelvis	143 (11.2%)	34,808 (2.0%)
Larynx	a	7580 (0.4%)
Leukemia	17 (1.3%)	62,942 (3.6%)
Lip Oral Cavity and Pharynx	53 (4.2%)	30,213 (1.7%)
Liver and Intrahepatic Bile Ducts	7 (0.5%)	28,907 (1.7%)
Melanoma of Skin	55 (4.3%)	42,909 (2.5%)
Mesothelioma	a	17,619 (1.0%)
Multiple Myeloma	27 (2.1%)	33,082 (1.9%
Non‐Hodgkin's Lymphoma	212 (16.7%)	69,079 (3.9%)
Esophagus	20 (1.6%)	58,461 (3.3%)
Other	224 (17.6%)	281,835 (16.1%)
Ovary	9 (0.7%)	35,299 (2.0%
Pancreas	19 (1.5%)	58,625 (3.3%)
Prostate	97 (7.6%)	212,824 (12.2%)
Stomach	22 (1.7%)	46,291 (2.6%)
Testis	6 (0.5%)	13,070 (0.7%)
Thyroid	16 (1.3%)	13,144 (0.8%)
Trachea Bronchus and Lung	90 (7.1%)	256,100 (14.6%)
Uterus	a	2688 (0.2%)
Total Cancers	1273 (100.0%)	1,750,197 (100.0%)

a Numerically too small to identify.

**Table 3:** Standardized incidence and mortality ratio after kidney transplantation

Type of cancer category	Cancer incidence (transplant vs. general population)	Cancer mortality (transplant vs. general population)	Noncancer mortality (transplant vs. general population)
	SIR (95% CI)	SMR (95% CI)	
Bladder	5.19 (4.91, 5.48)	0.76 (0.72, 0.80)	a
Brain	1.03 (0.97, 1.08)	0.63 (0.59, 0.66)	a
Breast	1.00 (0.95, 1.06)	0.97 (0.92, 1.02)	0.54 (0.51, 0.57)
Cervix Uteri	1.84 (1.74, 1.94)	0.40 (0.38, 0.43)	a
Colon and Rectum	1.49 (1.41, 1.58)	0.62 (0.59, 0.65)	1.06 (1.00, 1.12)
Hodgkin's Disease	3.23 (3.06, 3.41)	a	a
Kidney except Renal Pelvis	6.49 (6.14, 6.85)	0.42 (0.39, 0.44)	0.84 (0.79, 0.89)
Larynx	a	1.87 (1.77, 1.97)	a
Leukemia	1.31 (1.24, 1.39)	0.89 (0.84, 0.94)	a
Lip Oral Cavity and Pharynx	2.85 (2.70, 3.01)	0.99 (0.94, 1.05)	a
Liver and Intrahepatic Bile Ducts	0.93 (0.88, 0.99)	1.03 (0.97, 1.09)	a
Melanoma of Skin	1.99 (1.88, 2.10)	2.33 (2.21, 2.46)	0.47 (0.45, 0.50)
Mesothelioma	a	1.02 (0.97, 1.08)	a
Multiple Myeloma	3.74 (3.54, 3.95)	0.76 (0.72, 0.80)	a
Non‐Hodgkin's Lymphoma	10.12 (9.57, 10.68)	1.00 (0.94, 1.05)	1.12 (1.06, 1.18)
Esophagus	1.55 (1.47, 1.64)	0.87 (0.82, 0.92)	a
Other	4.84 (4.57, 5.10)	0.85 (0.81, 0.90)	1.54 (1.46, 1.63)
Ovary	0.92 (0.87, 0.97)	1.53 (1.45, 1.61)	a
Pancreas	1.65 (1.56, 1.74)	0.65 (0.61, 0.69)	a
Prostate	1.20 (1.13, 1.27)	0.64 (0.61, 0.68)	0.83 (0.79, 0.88)
Stomach	2.88 (2.72, 3.04)	0.80 (0.76, 0.85)	a
Testis	0.85 (0.80, 0.90)	a	a
Thyroid	2.23 (2.11, 2.35)	a	a
Trachea Bronchus and Lung	1.69 (1.60, 1.79)	0.88 (0.83, 0.93)	1.49 (1.41, 1.58)
Uterus	a	a	a
All	2.47 (2.34, 2.61)	0.75 (0.71, 0.79)	0.90 (0.85, 0.95)

a Numerically too small to identify.

**Table 4:** Cox regression analysis of time to cancer comparing transplant and general population cohort

Variable		Hazard ratio (95% CI)	*P*‐value
Age		1.00 (1.00–1.01)	0.012
Sex	Male	1 (baseline group)	
	Female	1.02 (0.94–1.10)	0.715
Ethnic group	White	1 (baseline group)	
	Black	1.16 (0.96–1.41)	0.121
	Other	1.04 (0.95–1.15)	0.367
Charlson comorbidity category	0	1 (baseline group)	
	1–4	0.96 (0.85–1.08)	0.510
	5+	1.07 (0.98–1.63)	0.142
Cohort	Transplant	1 (baseline group)	
	General population	1.04 (0.96–1.14)	0.299

In a Cox proportional hazards model for cancer incidence post–kidney transplantation, we observed increasing age to be strongly associated with the development of post‐transplant cancer (hazard ratio 1.05, 95% CI: 1.04–1.05). Other significant risk factors included recipients of deceased kidney donors (hazard ratio 1.14, 95% CI: 1.00–1.30) and increased number of readmissions (greater than three) within the first year of transplant (hazard ratio 3.58, 95% CI: 2.79–4.58). Repeat kidney transplant recipients were at reduced risk of developing post‐transplant cancer (hazard ratio 0.62. 95% CI: 0.46–0.85).

### Mortality risk after development of cancer

Mortality risk for the entire cohort after kidney transplantation was 2.83% (*n* = 562) and 12.38% (*n* = 2462) after 1 year and at any point during follow‐up, respectively. Among the 1273 patients who developed post‐transplant cancer (median time post‐transplant 3.8 years [interquartile range 1.8—6.2 years]), risk for mortality within 1 year and at any point during follow‐up was 3.61% and 41.95%, respectively.

Table 3 shows the respective standardized mortality rates for the kidney transplant population compared to the general population after a diagnosis of cancer. Despite increased incidence, risk for cancer‐related mortality was actually lower for kidney allograft recipients versus the general population after development of cancer (standardized mortality ratio 0.75, 95% CI: 0.71–0.79). In addition, noncancer‐related mortality was also lower for kidney allograft recipients versus the general population after diagnosis of cancer (standardized mortality ratio 0.90, 95% CI: 0.85–0.95). While these ratios varied dependent upon cancer site, the overall risk for cancer and noncancer‐related mortality was lower for kidney allograft recipients versus the general population.

## Discussion

In this retrospective population cohort study, we have compared cancer incidence and progression to mortality for kidney allograft recipients compared to the general population using hospital administrative data. In line with expectations, we observed an increased incidence of cancer after kidney transplantation. However, our data paradoxically observed reduced risk for both cancer‐related and noncancer‐related mortality after development of cancer for kidney allograft recipients versus the general population. The results of our study, which contradict published literature of increased cancer‐linked mortality after transplantation, question the use of hospital administrative data alone for cancer epidemiology studies and its associated methodology.

Cancer rates are known to increase over the spectrum of kidney disease but are significantly augmented after kidney transplantation 8. This is predominantly due to the contributing risk from immunosuppression, with incidence of cancer almost mirroring those observed in HIV/AIDS patients 2. Previous registry data from the United Kingdom highlighted double the risk for new‐onset cancer for transplant recipients versus the general population 12, and our analysis also confirms this increased risk for cancer after kidney transplantation. Recent work from Acuna and colleagues, using data linkage between Canadian Organ Replacement Register, the Ontario Cancer Registry, and the Office of the Registrar General of Ontario, also showed increased risk for cancer‐specific death for solid organ transplant recipients versus the general population in Ontario regardless of age, sex, or transplanted organ 13. Our current findings even contradict our previous work, also using hospital administrative data, which demonstrated increased risk for cancer‐related mortality for kidney allograft recipients versus the general population in England 5.

However, there are methodological differences between these studies and our current analysis. Previous work explored risk for cancer‐related mortality among all kidney allograft recipients, while our current analysis examines risk for cancer‐related mortality among kidney allograft recipients who *specifically* developed cancer after transplantation. This is an important difference because, being aware that cancer incidence is significantly increased after kidney transplantation, analyzing cancer‐related mortality as a proportion of the entire kidney allograft cohort will skew the standardized incidence ratio to imply higher cancer‐related mortality after kidney transplantation versus the general population. Standardized cancer mortality ratios were observed to be similar between kidney transplant and general population cohorts by Kiberd and colleagues, in their analysis of the United States Renal Data System (study period January 1990 to December 2004) 5. The authors suggested cancer‐related mortality rates were similar due to possible competing risks for death after kidney transplantation but failed to explore this further. Our study explores this for the first time and demonstrates a significantly lower risk for noncancer‐related mortality after development of cancer for kidney allograft recipients versus the nontransplant population. We can speculate that cancer is diagnosed earlier for kidney allograft recipients, due to their close lifelong supervision under the specialist care of nephrologists. Supporting this notion is data from Cho and colleagues, analyzing the Surveillance, Epidemiology, and End Results (SEER) program, who observed noncancer‐related survival was higher for patients diagnosed with early cancer versus the matched US general population, while the reciprocal was true for advanced cancer 20. This would fit in with the observation from Table 3, which suggests the most incident post‐transplant cancers appear to have the greater survival benefit. However, identifying less noncancer‐related mortality for kidney transplant recipients who develop cancer seems implausible considering the burden of complications associated with long‐term immunosuppression.

The progressive risk of cancer from incidence to mortality has been reported in the general population. Jemal et al. 21 reviewed cancer incidence and mortality from global population‐based cancer incidence data and observed disparate epidemiological patterns. While cancer rates for malignancies commonly attributed to developed countries (e.g., lung, colorectal, breast) are falling, their rates are increasing in the developing world. In addition, infection‐related cancers (e.g., cervical, liver, digestive organs) continue to disproportionately affect developing countries. This latter point is important for our study as cancers secondary to an infectious etiology predominate after kidney transplantation, with kidney cancers the exception. This was shown in the study from Grulich and colleagues, where cancer incidence for kidney allograft recipients was broadly on a par with comparable immune deficiency states such as HIV/AIDS 2. The etiology of cancer post‐transplantation is primarily driven by the effects of immunosuppression, with suggestions of an important interplay with viral pathogens that contribute to pathophysiology 22. While we did not have access to immunosuppression regimen data in our analysis, recent data would suggest similar carcinogenicity among different immunosuppressant regimens 23 meaning there should be minimal confounding from different immunosuppression regimens in use across England.

The likeliest explanation for our data is the inaccuracy of cancer‐related outcomes in hospital administrative data. However, the paradoxical data in our analysis reflect the standardized mortality ratio rather than the incidence ratio. We would anticipate the latter being underrepresented by hospital administrative data as only hospitalized data would be captured. Therefore, cancers not requiring hospital admission (e.g., little treatment potential or outpatient management sufficient) will not be captured. However, mortality events should be robustly captured using linkage of the Hospital Episode Statistics to the Office for National Statistics which capture all deaths. Therefore, our paradoxical findings of lower cancer and noncancer mortality events among kidney transplant recipients seem puzzling. While contradictory to published literature and registry reports, there could be some plausible explanations for our findings. Cancer survival may be better due to earlier diagnosis after kidney transplantation due to close supervision and heightened risk awareness. In addition, cancer management after kidney transplantation frequently involves tapering of immunosuppression, which may be more effective than conventional treatment alone at attenuation of cancer‐related mortality. Reduced noncancer mortality may be secondary to aggressive medical management by transplant clinicians, with patients remaining under lifelong surveillance and supervision of secondary care specialists. However, increased cardiovascular‐related mortality is generally noted for kidney allograft recipients versus the general population 24 and reasserts the paradoxical nature of our results. Kidney allograft recipients may be more compliant with national screening protocols, although robust evidence‐base for optimal screening protocols are lacking 25, 26, and recent evidence from a Canadian transplant cohort in fact suggests kidney transplant recipients have worse adherence to cancer screening programs compared to the general population 27.

The linkage of the large HES administrative dataset of secondary care to official mortality data held by ONS ensures generalizability and applicability to the wider transplant community. While this ensures the robustness of our findings, we have several limitations to acknowledge which may influence our results. The biggest limitation is that cancer registration will be underestimated using HES data rather than dedicated cancer registry data as it only identifies patients hospitalized with cancer codes. This is consistent with a study from the United States showing cancer information from administration data is substantially incomplete in relation to cancer registries 28. Administrative and registry data have well‐documented limitations, both generally 29 and in the context of solid organ transplantation 14, that lead to inherent problems with such analyzes. There will be significant confounding from lead‐time bias, as first documented cancer episode may not necessarily represent the initial cancer diagnosis with a false impression of survival subsequently assumed. From a HES perspective, our analysis of the cancer incidence ratio is skewed against cancers not likely to lead to a hospitalization episode (i.e., nonmelanoma skin cancers which are the most commonly occurring cancers after kidney transplantation). We therefore opted to omit all nonmelanoma skin cancer from our analysis as they are least likely to cause death 5, 6, 13 and will have little impact upon our mortality observations. The hospitalized general population cohort could have a greater burden of health comorbidities that has skewed comparisons with kidney transplant recipients, leading to the paradoxical survival outcomes for the latter. We attempted to correct for the general population confounder by calculating standardized incidence and mortality ratios of the hospitalized general population cohort using life‐table ratios for the adult general population. The lack of information on renal and transplant‐specific factors, available from the UK Renal and Transplant Registry, respectively, is important as they can impact upon cancer‐related epidemiology (e.g., dialysis vintage, kidney rejection and/or failure). A deeper probe of this cohort will require analysis of cancer stages, grading and management (available from cancer registries) and linkage to primary care data resources to obtain information regarding prescriptions (especially immunosuppression). The Epidemiology of Cancer after Solid Organ Transplantation (EpCOT) project, by facilitating record linkage between these different national registries (e.g., transplant, cancer, hospital administration, mortality) for all solid organ transplant recipients in England, should provide a more comprehensive analysis to answer the unanswered questions from this analysis (clinicaltrials.org identifier—NCT02991105).

To conclude, our population cohort study of kidney allograft recipients in England has demonstrated an increased risk for developing cancer compared to the general population consistent with the published literature. However, after development of cancer, our data suggest kidney allograft recipients have reduced risk for both cancer and noncancer‐related mortality compared to the general population. This paradoxical finding is unlikely to be plausible and suggests limitations with relying upon hospital administrative data for cancer‐related epidemiological studies. The limitations from our analysis suggest comprehensive studies on this issue will require robust record linkage between numerous data registries to truly establish our understanding of the risk of mortality for kidney transplant recipients after they develop cancer.

## Conflict of Interest

None declared.
